# Graded hypergravity is associated with large-scale reorganization of resting-state cortical networks: EEG evidence from a neurological inactivity phenotype

**DOI:** 10.1016/j.ibneur.2026.06.016

**Published:** 2026-06-22

**Authors:** Chrysoula Kourtidou-Papadeli, Christos Giantsios, Christos Frantzidis, Ilias Machairas, Sofia Kourtidou, Panagiotis D. Bamidis, Joan Vernikos

**Affiliations:** a Laboratory of Medical Physics and Digital Innovation, School of Medicine, Faculty of Health Sciences, Aristotle University of Thessaloniki, Thessaloniki, Greece; b Greek Aerospace Medical Association and Space Research (GASMA-SR), Thessaloniki, Greece; c Aeromedical Center of Thessaloniki (AeMC), Kalamaria, Greece; d Laboratory of Biochemistry, Department of Medicine, School of Health Sciences, Aristotle University of Thessaloniki, Greece; e School of Engineering and Physical Sciences, University of Lincoln, Brayford Pool, LN6 7TS, UK; f Thirdage LLC, Culpeper, VA, United States

**Keywords:** Keywords: Hypergravity, Resting-state EEG, Functional connectivity, Graph-theoretical analysis, Cortical network plasticity

## Abstract

**Background:**

Chronic physical inactivity and reduced sensorimotor engagement are associated with alterations in large-scale brain network organization, often reflected as changes in resting-state functional connectivity. Interventions capable of modulating multisensory input without requiring volitional movement may therefore provide a useful experimental framework for investigating inactivity-related cortical plasticity. Short-arm human centrifugation (SAHC) delivers controlled, intermittent graded hypergravity, providing a reproducible vestibular–somatosensory stimulus with potential neuromodulatory effects on cortical networks.

**Methods:**

In this exploratory pilot study, resting-state electroencephalography (EEG) was recorded before and after repeated SAHC exposure in a neurological inactivity cohort predominantly comprising individuals with multiple sclerosis. EEG signals were reconstructed at the cortical source level using standardized low-resolution electromagnetic tomography (sLORETA) and parcellated into 148 cortical regions. Functional connectivity was estimated using synchronization likelihood, and large-scale network organization was characterized using graph-theoretical analysis. Network-Based Statistics (NBS) was applied to identify statistically significant pre–post connectivity differences.

**Results:**

Following repeated exposure to graded hypergravity, 1333 cortical connections exhibited statistically significant changes, all reflecting reductions in resting-state functional connectivity. These effects were distributed across widespread cortical regions, indicating large-scale network reorganization rather than focal modulation. The intervention was well tolerated, and no serious adverse events were reported.

**Conclusions:**

Graded hypergravity delivered via short-arm human centrifugation was associated with widespread reorganization of resting-state cortical functional networks in a phenotype of prolonged inactivity. The observed reductions in functional connectivity are consistent with adaptive network reconfiguration. These findings suggest that controlled multisensory gravitational stimulation can modulate large-scale cortical network organization under conditions of prolonged inactivity. Given the small and heterogeneous pilot cohort, these findings should be considered exploratory and hypothesis-generating.

## 1 Introduction

Prolonged physical inactivity, whether arising from neurological disease, immobilization, or environmental constraints, induces multisystem deconditioning that affects musculoskeletal, cardiovascular, vestibular, and neural function. Reduced motor output and diminished sensorimotor engagement disrupt physiological homeostasis and drive maladaptive plastic changes across multiple organ systems, including the central nervous system (Vernikos, 1996, Clément and Pavy-Le Traon, 2004, Hargens and Vico, 2016). Similar deconditioning phenomena are consistently observed in chronic neurological conditions, where reduced mobility and altered sensory input contribute to progressive functional decline and impaired neural efficiency.

Multiple sclerosis (MS) exemplifies a condition in which neurological pathology is accompanied by prolonged physical inactivity and altered sensorimotor integration. Beyond inflammation-induced demyelination and axonal damage, increasing evidence indicates that MS is associated with large-scale reorganization of functional brain networks, extending well beyond focal lesions (Filippi et al., 2013, Rocca et al., 2018, Qu et al., 2026). Resting-state neuroimaging studies have frequently reported abnormal increases in functional connectivity and disrupted network integration, a pattern often interpreted as compensatory but metabolically inefficient hyperconnectivity (Stam, 2014, Hillary and Grafman, 2017). Over time, such network-level alterations may impose increased energetic cost and contribute to fatigue, cognitive impairment, and reduced functional reserve.

Physical activity and mechanical loading are increasingly recognized as potent modulators of brain plasticity and large-scale network organization. Exercise has been shown to promote neuroprotection, enhance synaptic efficiency, and facilitate adaptive cortical reorganization in both healthy individuals and neurological populations (Bullmore and Sporns, 2012, Erickson et al., 2019). However, conventional exercise-based interventions often require voluntary effort, balance, and mobility that may not be feasible for individuals with advanced neurological disability. This limitation highlights the need for alternative strategies capable of delivering multisystem stimulation and neural engagement with minimal volitional demand.

Short-arm human centrifugation delivers graded, intermittent hypergravity and is best conceptualized as a controllable gravitational loading stimulus rather than a full gravity replacement. By providing externally imposed mechanical loading in a supine position, short-arm human centrifugation simultaneously stimulates vestibular, proprioceptive, cardiovascular, and musculoskeletal systems, reproducing key physiological aspects of upright posture and whole-body activity (Clément et al., 2015, Hargens and Vico, 2016). Recent experimental evidence demonstrates that hypergravity exposure induces functional and molecular adaptations within vestibular pathways, including changes in vestibular receptor expression and vestibulo-ocular responses (Choi et al., 2025). Dose–response studies have demonstrated that intermittent exposure to graded hypergravity can elicit physiological and autonomic responses comparable to those observed during standing and light-to-moderate exercise, supporting its potential as a non-volitional, multisensory intervention (Kourtidou-Papadeli et al., 2021).

Beyond its peripheral effects, emerging evidence suggests that graded hypergravity may exert neuromodulatory influences on brain function. In a recent clinical study, short-arm human centrifugation applied to a patient with secondary progressive multiple sclerosis resulted in measurable improvements in disability, balance, cardiovascular performance, and cortical functional connectivity, consistent with adaptive neuroplastic reorganization (Kourtidou-Papadeli et al., 2022). These observations support the view that gravitational loading constitutes a systems-level stimulus capable of reshaping large-scale brain networks, rather than acting solely through peripheral mechanical mechanisms.

In this context, the present study investigates whether repeated exposure to graded hypergravity delivered via short-arm human centrifugation induces adaptive reorganization of cortical functional networks in a phenotype of prolonged inactivity. By applying graph-theoretical analysis to source-level resting-state electroencephalographic connectivity, this work aims to elucidate how externally imposed gravitational loading influences network efficiency, information transfer, and energetic organization in the human brain (Frantzidis et al., 2019, Chriskos et al., 2018). Understanding these mechanisms may advance the development of non-volitional neuromodulatory approaches for neurorehabilitation and provide broader insight into the principles governing inactivity-related brain network plasticity.

## 2 Materials and methods

### 2.1 Study aim and analytical framework

The purpose of the present analysis was to investigate alterations in brain functional connectivity before and after a repeated short-arm human centrifugation (SAHC) intervention in a phenotype of prolonged neurological inactivity, predominantly comprising individuals with multiple sclerosis. Specifically, we examined whether intermittent exposure to graded hypergravity delivered via SAHC is associated with neuroplastic changes in large-scale cortical networks under conditions of reduced sensorimotor engagement.

To this end, functional connectivity was estimated from electroencephalographic (EEG) rhythmic activity at the cortical source level. Brain networks were constructed using functional connectivity measures, and their organizational properties were quantified using graph-theoretical analysis.

### 2.2 Short-arm human centrifuge infrastructure

Graded hypergravity delivered by SAHC constitutes a controlled multisystem stimulus engaging vestibular, proprioceptive, cardiovascular, musculoskeletal, and sensorimotor pathways. The SAHC device developed by our research group (Patent No. 1009812/13–09–2019) is located at the “Joan Vernikos” Laboratory of Aerospace and Rehabilitation Applications. The centrifuge has a diameter of 4 m and can generate up to + 3.5 Gz at the feet. It consists of a central axis supporting two to four beds mounted on smooth-rolling wheels, driven by a motor–reducer system.

The infrastructure includes exercise systems mounted at the extremities of the beds, allowing aerobic training via a cycle ergometer and resistance training via a horizontal rowing device. The entire system is operated through an electronic control panel and was constructed in compliance with national and international safety standards, making it suitable for individuals with mobility impairments.

### 2.3 Participants and experimental design

Data were derived from experiments conducted by the Laboratory of Medical Physics and Digital Innovation of the Aristotle University of Thessaloniki in collaboration with the Greek Aerospace Medical Association - Space Research (GASMA-SR) and the Rehabilitation Center of “Arogi”. The cohort consisted of eight participants: five with multiple sclerosis, one with motor neuron syndrome, one with stroke, and one with a neurodevelopmental disorder. Although the cohort included individuals with different neurological conditions, participants were selected based on a shared phenotype of prolonged sensorimotor inactivity rather than on disease-specific mechanisms. Given the exploratory nature of the study and the rarity of the phenotype, the sample size reflects a pilot investigation intended to identify large-scale network effects. All participants underwent a 10 min familiarization centrifugation trial at 1 G and were included after demonstrating tolerance without dizziness or nausea. Centrifugation began at 0.5 G and progressed through 0.7, 1.0, 1.2, 1.5, 1.7, and 2.0 G, each lasting 5 min with gradual acceleration/deceleration and 6-min rest intervals. Total session duration was approximately 90 min.

The participants were secured with safety harnesses, and to minimize Coriolis effects, the head was positioned near the center of rotation.

### 2.4 EEG data acquisition

Resting-state EEG recordings were acquired under eyes-closed resting conditions. EEG was recorded using a Neurofax EEG−1200 system (Nihon Kohden, Tokyo, Japan) with 19 Ag/AgCl electrodes placed according to the international 10–20 system. Signals were sampled at 500 Hz.

Electrooculography (EOG), electrocardiography (ECG), and electromyography (EMG) were simultaneously recorded to assist artifact identification. A ground electrode was placed at Fpz, and reference electrodes were placed on the mastoids.

### 2.5 EEG preprocessing

EEG signals were re-referenced to the common average reference. Filtering included: high-pass filter at 0.5 Hz, low-pass filter at 50 Hz, and notch filters at 50 and 100 Hz.

Independent Component Analysis (ICA) was applied to remove ocular, muscular, and cardiac artifacts using custom Python scripts and visual inspection in MATLAB/EEGLAB. Artifact-free data were segmented into non-overlapping epochs of 16 s.

### 2.6 Source reconstruction and functional connectivity

Source-reconstructed cortical activity was represented by approximately 15,000 cortical dipoles constrained to the cortical surface and grouped into 148 anatomical regions according to the Destrieux atlas (Pascual-Marqui, 2002). Regional time series were obtained by averaging dipole activity within each region. Functional connectivity between cortical regions was then estimated using synchronization likelihood, a nonlinear measure robust to noise and non-stationarity (Stam and van Dijk, 2002), yielding weighted connectivity matrices for each participant and condition. Graph-theoretical analysis was performed on these matrices as described below.

### 2.7 Graph-theoretical network analysis

Functional connectivity matrices were converted into undirected, binary graphs by retaining the top 20% of connectivity values using an adaptive threshold to ensure equal network density across participants and conditions, following standard practices in brain network analysis (Rubinov and Sporns, 2010, Lozano-Bagén et al., 2025). Global network metrics, including clustering coefficient, characteristic path length, global efficiency, and modularity, were computed using functions from the Brain Connectivity Toolbox (Rubinov and Sporns, 2010). Betweenness centrality was calculated to identify network hubs.

### 2.8 Statistical analysis

Network-Based Statistics (NBS) was employed to identify statistically significant pre–post differences in functional connectivity. Paired *t*-tests were applied within the NBS framework for pre–post comparisons. An exploratory test-statistic threshold analysis (range 0.5–3.0, step 0.1) identified t = 1.8 as the optimal primary threshold. Statistical significance was assessed using permutation testing with family-wise error correction (p < 0.05, two-tailed).

## 3 Results

### 3.1 Large-scale cortical connectivity changes

At the group level across participants, Network-Based Statistics revealed a total of 1333 cortical connections exhibiting statistically significant differences between pre- and post-intervention conditions following the SAHC intervention (NBS, p < 0.05, family-wise error corrected). All significant effects corresponded to reductions in resting-state functional connectivity, with no statistically significant increases observed.

**Fig. 1:**
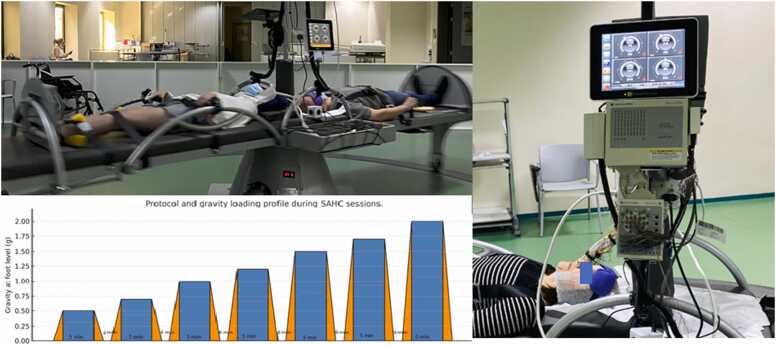
Schematic representation of the short-arm human centrifugation (SAHC) protocol, including the centrifuge system, the centrifugation protocol, the EEG recording device, and the cardiovascular monitoring equipment.

**Fig. 2:**
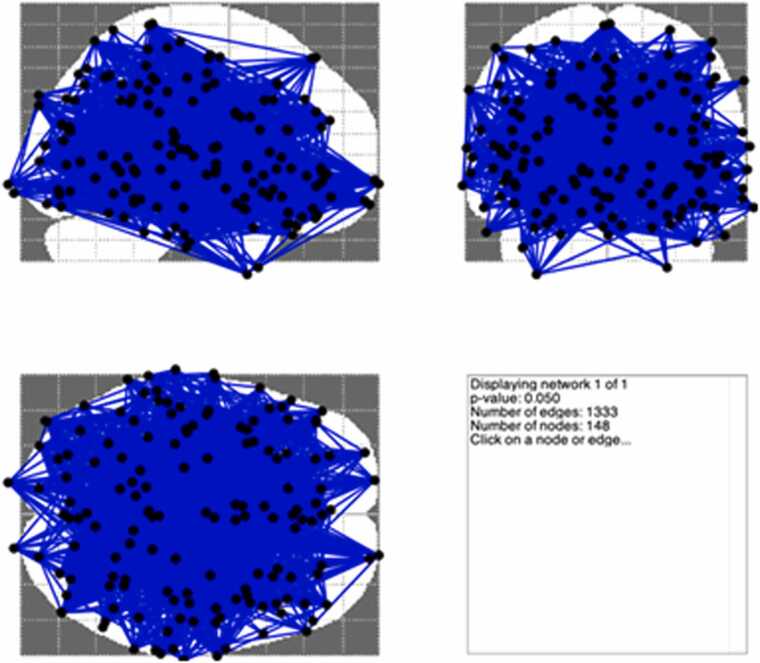
Cortical connections showing statistically significant reductions in resting-state functional connectivity after SAHC, identified by Network-Based Statistics (family-wise error corrected, p < 0.05). Effects are distributed across widespread cortical regions.

**Fig. 3:**
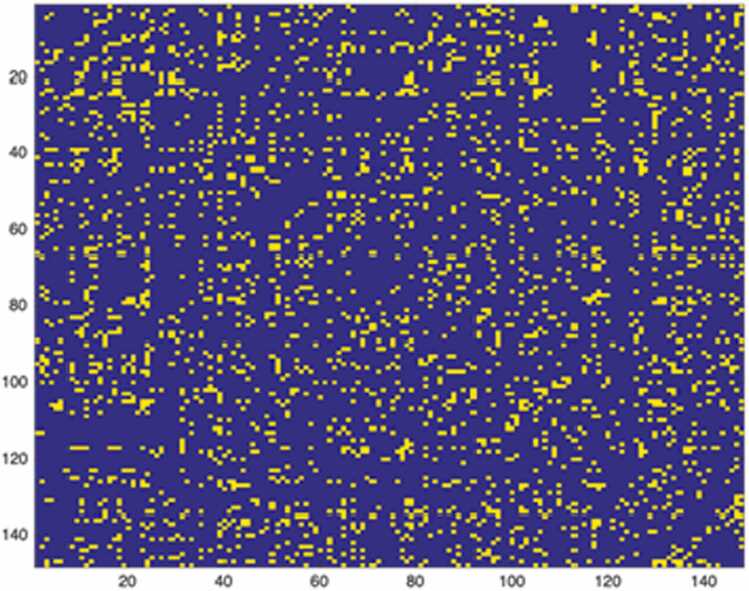
Binary adjacency matrix of statistically significant functional connectivity reductions identified by Network-Based Statistics. Entries indicate significant (1) or non-significant (0) connections; 1333 cortical connections showed reduced connectivity after centrifugation.

The affected connections were distributed across widespread cortical regions, indicating large-scale changes in functional network organization rather than localized or region-specific effects. Visualization of the resulting subnetwork (Fig. 1, Fig. 2) shows that the identified connectivity reductions involved distributed cortical systems spanning frontal, parietal, temporal, and occipital regions. The corresponding adjacency matrix (Fig. 3) illustrates the extent and topology of the affected network.

### 3.2 Network topology of the affected subnetwork

Graph-theoretical analysis was performed on the subnetwork consisting exclusively of statistically significant edges identified by Network-Based Statistics. This subnetwork exhibited a density of 0.1178, reflecting a sparse pattern of retained connections among the affected edges.

The mean clustering coefficient was 0.1097, indicating limited local clustering among interconnected cortical regions. The characteristic path length was 0.1178, reflecting relatively short communication paths among nodes within the subnetwork.

The modularity index was Q = 0.1309, indicating weak but detectable modular organization, while global efficiency was E = 0.5207, reflecting preserved efficiency of information transfer within the identified subnetwork.

### 3.3 Identification of functional hubs

**Fig. 4:**
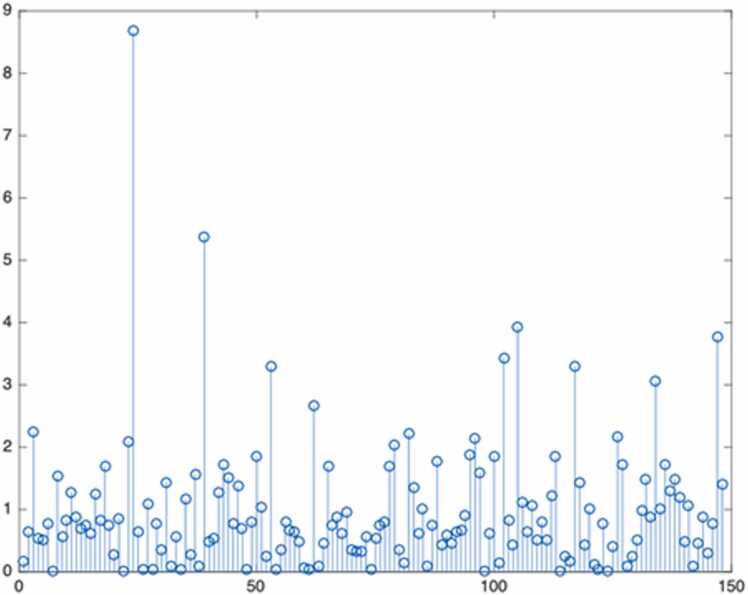
Cortical regions identified as functional hubs based on betweenness centrality analysis of the significant network. Nodes with betweenness centrality ≥ 1.5 were classified as hubs, indicating a key role in information transfer.

Betweenness centrality analysis identified multiple cortical regions with betweenness centrality values Bi ≥ 1.5, which were classified as functional hubs within the post-intervention subnetwork. These hubs were distributed across frontal, parietal, temporal, and occipital cortices (Fig. 4).

The cortical regions corresponding to the identified hubs are summarized in Table 1. The distribution of hubs across multiple lobes indicates that the statistically significant connectivity changes involved nodes spanning multiple large-scale cortical systems.

**Table 1:** Brain regions that are matched to functional hubs according to the Destrieux atlas.

Hubs	Brain Regions
3	Left Limbic Gyrus and Sulcus cingul-Ant
8	Right Limbic Gyrus and Sulcus cingul-Mid-Post
18	Right PreFrontal Gyrus and Sulcus transv frontopol
23	Left Occipital Gyrus cuneus
24	Right Occipital Gyrus cuneus
37	Left Temporal Gyrus oc-temp lat-fusifor
39	Left Occipital Gyrus oc-temp med-Lingual
43	Left Occipital Gyrus occipital middle
44	Right Occipital Gyrus occipital middle
50	Right Parietal Gyrus pariet inf-Angular
53	Left Parietal Gyrus parietal sup
62	Right PreFrontal Gyrus rectus
65	Left Temporal Gyrus temp sup-G transv
78	Right Frontal Lat Fis-Ant-Horizont
79	Left Frontal Lat Fis-Ant-Vertical
82	Right Temporal Lat Fis-post
88	Right Occipital Sulcus calcarine
95	Left Temporal Sulcus circular insula inf
96	Right Temporal Sulcus circular insula inf
97	Left Temporal Sulcus circular insula sup
100	Right Temporal Sulcus collat transv ant
102	Right Occipital Sulcus collat transv post
105	Left Frontal Sulcus front middle
113	Left Temporal Sulcus oc-temp lat
117	Left Occipital Sulcus oc middle and Lunatus
126	Right PreFrontal Sulcus orbital lateral
127	Left PreFrontal Sulcus orbital med-olfact
134	Right C Sulcus postcentral
136	Right C Sulcus precentral-inf-part
147	Left Temporal Sulcus temporal transverse

## 4 Discussion

### 4.1 Large-scale network reorganization following SAHC

In the present study, repeated exposure to graded hypergravity delivered via short-arm human centrifugation (SAHC) was associated with statistically significant alterations in 1333 resting-state cortical connections in a phenotype of prolonged neurological inactivity, predominantly comprising individuals with multiple sclerosis. Reduced resting-state connectivity does not necessarily reflect impairment, but may indicate normalization of hyperconnective compensatory patterns observed in neurological conditions.

Previous studies have shown that neurological and neurodegenerative conditions are frequently characterized by abnormal increases in resting-state connectivity, often interpreted as compensatory responses to structural or functional disruption (Stam, 2014, Hillary and Grafman, 2017). However, such hyperconnectivity may reflect inefficient network states associated with increased metabolic cost and reduced functional reserve (Piervincenzi et al., 2025). Within this framework, the widespread connectivity reductions observed following SAHC may reflect attenuation of maladaptive resting-state coupling rather than loss of functional integration.

### 4.2 Network efficiency and information transfer

Graph-theoretical analysis indicated that the subnetwork of altered connections retained short characteristic path lengths and moderate global efficiency, despite the overall reduction in functional connectivity. Short path lengths and preserved efficiency are key features of effective information transfer in large-scale brain networks (Watts and Strogatz, 1998, Rubinov and Sporns, 2010). In neurological conditions associated with prolonged inactivity and impaired sensorimotor integration, disruptions in these parameters have been linked to reduced cognitive and motor performance (Stam et al., 2007).

Accordingly, the post-intervention network configuration observed here is compatible with preserved capacity for information transfer despite reduced overall coupling. While the present data do not permit direct inference regarding behavioral or cognitive outcomes, the observed topological features align with models in which reductions in resting-state hyperconnectivity reflect adaptive redistribution of network resources rather than functional degradation.

### 4.3 Resting-state hyperconnectivity and energetic cost

In multiple sclerosis and related neurological conditions, increased resting-state cortical activity and connectivity have been widely reported and interpreted as compensatory mechanisms in response to structural damage or impaired conduction (Filippi et al., 2013, Rocca et al., 2018). However, persistent hyperactivation may impose a substantial energetic burden on neural systems already compromised by disease, potentially contributing to fatigue and progressive functional decline (DeLuca et al., 2008, Broeders et al., 2024).

Within this context, the connectivity reductions observed following SAHC are compatible with attenuation of energetically costly resting-state coupling. Although this interpretation remains indirect, it is supported by converging evidence from network neuroscience indicating that excessive synchronization is not synonymous with improved network performance and may, in fact, reflect maladaptive organization (Achard and Bullmore, 2007, Stam, 2014).

### 4.4 Distributed hubs and large-scale integration

Betweenness centrality analysis identified functional hubs distributed across frontal, parietal, temporal, and occipital cortices. Similar large-scale alterations of network architecture and hub organization have recently been reported in multiple sclerosis using multiplex network approaches (Pontillo et al., 2025).

Importantly, the preservation of hub structure within the reorganized network supports the interpretation that SAHC-induced changes reflect reconfiguration rather than disruption of cortical functional architecture. This finding aligns with models of brain network plasticity emphasizing flexible redistribution of connectivity while maintaining core integrative properties.

### 4.5 Neuroplastic interpretation of SAHC-induced network changes

The widespread reorganization of cortical functional connectivity observed following SAHC is consistent with functional neuroplasticity at the level of large-scale brain networks. In the present context, neuroplasticity refers to experience-dependent reorganization of functional interactions among distributed cortical regions, inferred from altered patterns of synchronization and information routing, rather than to synaptic or structural plasticity per se (Bullmore and Sporns, 2012, Stam, 2014).

Functional brain networks are dynamic and continuously shaped by sensory input, motor activity, and autonomic state (Deco et al., 2011). Alterations in gravitational loading constitute a potent multisensory perturbation, simultaneously engaging vestibular, somatosensory, proprioceptive, and cardiovascular systems (Angelaki and Cullen, 2008, Lackner and DiZio, 2005). Repeated exposure to such stimuli, as delivered by SAHC, provides a plausible substrate for experience-dependent reweighting of large-scale cortical networks. This interpretation is supported by recent experimental evidence showing hypergravity-induced adaptations in vestibular nuclei and receptor expression, indicating that altered gravitational loading can drive neuroplastic processes throughout the vestibular system (Choi et al., 2025).

Within neurological inactivity phenotypes, including multiple sclerosis, resting-state hyperconnectivity has frequently been reported and interpreted as compensatory. However, accumulating evidence suggests that such hyperconnectivity may reflect an energetically inefficient configuration associated with reduced functional reserve (Achard and Bullmore, 2007, Hillary and Grafman, 2017). In this framework, the reductions in functional connectivity observed following SAHC are compatible with attenuation of maladaptive coupling rather than loss of functional integration.

### 4.6 Translational implications

Beyond its relevance to neurological inactivity, the present findings highlight the capacity of controlled multisensory gravitational stimulation to modulate large-scale brain network organization. Short-arm human centrifugation offers a unique experimental approach for delivering externally imposed multisensory gravitational stimulation and has been increasingly recognized as a promising artificial-gravity countermeasure with applications in rehabilitation and human spaceflight (Goswami et al., 2026). Such approaches may inform the development of novel neuromodulatory strategies for neurorehabilitation and provide insight into general principles governing inactivity-related brain network reorganization.

### 4.7 Limitations

Several limitations of the present study should be acknowledged. First, the sample size was small and heterogeneous. Given the exploratory nature of this pilot investigation and the rarity of the phenotype, the sample size was intended to detect large-scale network effects rather than condition-specific differences. Consequently, disease-specific conclusions cannot be drawn, and the results should be considered exploratory and hypothesis-generating. Although this limits statistical generalizability, the consistency and magnitude of the observed network changes support the robustness of the primary findings and motivate further investigation in larger and more homogeneous cohorts.

Second, the absence of a control group limits causal inference regarding the observed connectivity changes.

Third, the analysis was restricted to the eyes-closed resting-state condition. Future studies should incorporate additional behavioral states, postural conditions, and task-based paradigms to better characterize the functional significance of the observed network reorganization and its relationship to cognitive and motor performance.

Fourth, source localization was performed using conventional-density EEG and a generic head model, which inherently limits spatial precision compared with high-density EEG combined with individual MRI-based reconstruction. The integration of high-density EEG with individual MRI-based source models and complementary neuroimaging modalities may allow more precise characterization of cortical and subcortical contributions to the observed effects. Randomized controlled studies in well-defined neurological inactivity cohorts, including individuals with multiple sclerosis, are warranted to confirm the reproducibility of SAHC-induced network changes and to establish dose–response relationships related to gravitational loading parameters and exposure duration.

Finally, although widespread reductions in functional connectivity were observed, the underlying neurobiological mechanisms remain speculative. Future randomized controlled studies with larger homogeneous cohorts, multimodal neuroimaging, and longitudinal follow-up are required to confirm the reproducibility, clinical significance, and mechanistic basis of SAHC-induced cortical network reorganization.

### 4.8 Conclusions

This study demonstrates that repeated exposure to graded hypergravity delivered via short-arm human centrifugation is associated with widespread reorganization of resting-state cortical functional networks in a phenotype of prolonged neurological inactivity, predominantly comprising individuals with multiple sclerosis. The observed reductions in functional connectivity across large-scale networks are compatible with adaptive functional reconfiguration, potentially reflecting attenuation of maladaptive resting-state hyperconnectivity and a shift toward a more economical network organization.

These findings support the concept that controlled multisensory gravitational loading can act as a neuromodulatory stimulus capable of reshaping large-scale brain network organization. Short-arm human centrifugation therefore represents a novel experimental platform for probing inactivity-related brain network plasticity and for exploring non-volitional neuromodulatory interventions in neurological populations. More broadly, the present results contribute to understanding how externally imposed sensory and mechanical stimuli influence large-scale functional brain organization under conditions of reduced sensorimotor engagement.

## CRediT authorship contribution statement

**Bamidis Panagiotis:** Validation, Supervision, Project administration. **Joan Vernikos:** Supervision. **Ilias Machairas:** Supervision, Formal analysis. **Sofia Kourtidou:** Methodology, Data curation. **Christos Frantzidis:** Validation, Supervision, Resources, Formal analysis. **Chrysoula Kourtidou-Papadeli:** Writing – review & editing, Visualization, Supervision, Conceptualization. **Christos Giantsios:** Writing – original draft, Methodology, Formal analysis, Data curation.

## Ethics statement

The study was conducted in accordance with the Declaration of Helsinki and was approved by the Bioethics Committee of the School of Medicine of the Aristotle University of Thessaloniki (Approval No. 179/19.03.2020). All participants were informed about the aims of the rehabilitation program and the experimental procedures and were allowed to ask questions before enrollment. Written informed consent was obtained from all participants before participation. The therapeutic intervention was registered in the ClinicalTrials.gov repository (Identifier: NCT04369976).

## Declaration of Competing Interest

The authors declare that they have no known competing financial interests or personal relationships that could have appeared to influence the work reported in this paper.
